# Multi-scale digital twins for personalized medicine

**DOI:** 10.3389/fdgth.2026.1753906

**Published:** 2026-02-16

**Authors:** Alexandre Vallée

**Affiliations:** Department of Epidemiology and Public Health, Foch Hospital, Suresnes, France

**Keywords:** artificial intelligence, biomedical data integration, causal inference, computational modeling, digital health, ethical challenges, graph neural networks, machine learning

## Abstract

The future of personalized medicine requires moving beyond isolated data streams toward integrated, multi-scale representations of human health. Digital twins (DTs) have emerged as promising solutions, offering dynamic, individualized simulations of biological systems. However, current implementations often rely on narrow data sources, limiting predictive power, adaptability, and clinical utility. The next generation of digital twins must integrate molecular, cellular, tissue, organ, clinical, behavioral, and environmental data to accurately model health trajectories and disease evolution. This review synthesizes the conceptual foundations, technical architectures, clinical applications, and ethical challenges associated with multi-scale digital twins (MSDTs). Key enabling technologies include multimodal data fusion, graph neural networks, causal inference frameworks, reinforcement learning, and hybrid mechanistic–AI modeling approaches. Clinical applications can illustrate the potential of MSDTs to personalize interventions dynamically. Significant barriers persist regarding data integration, ethical governance, bias mitigation, and regulatory adaptation.

## Introduction

The future of personalized medicine requires a paradigm shift, including moving beyond isolated data streams toward integrated and multi-scale representations of human health (1). Digital twins (DTs) have emerged as a promising technological solution, offering dynamic, individualized simulations of biological systems (2, 3). However, most current digital twin implementations are constrained to narrow data sources, often relying exclusively on electronic health records, imaging, or single-omics profiles (4). This siloed approach limits their predictive power, adaptability, and clinical utility (5).

This work argues that the next generation of digital twins must embrace a multi-scale framework, encompassing molecular (genomic, transcriptomic, proteomic, metabolomic), cellular, tissue, organ, clinical, behavioral, and environmental dimensions (6, 7). Only through the integration of these diverse data layers can digital twins truly capture the complexity of human health trajectories, disease evolution, and therapeutic responses (8).

Such multi-scale digital twins (MSDTs) would not merely forecast likely outcomes; they would simulate alternative futures (9), optimize intervention strategies (10), and dynamically adapt to the evolving biological and environmental context of each patient (11). MSDTs represent the convergence of systems biology, artificial intelligence, and clinical medicine into a unified, actionable model of personalized health (12).

This work advocates for a coordinated, interdisciplinary effort to develop multi-scale digital twins, addressing key methodological, ethical, and infrastructural challenges (13). This work argues that without embracing multi-scale integration, digital twins will remain limited tools, failing to realize their full transformative potential in medicine (14). This review was designed as a structured narrative synthesis focusing on representative and methodologically foundational contributions rather than a fully exhaustive systematic review.

### Methodology

A structured literature search was conducted across PubMed, Scopus, and Web of Science from database inception to March 2025 using the following query: (“digital twin” OR “digital twins”) AND (“multi-scale” OR “multi-omics” OR “systems biology” OR “hierarchical modeling”) AND (“personalized medicine” OR “precision medicine” OR “individualized healthcare”).

### Eligibility criteria

#### Inclusion criteria

Articles were eligible if they:

1. addressed digital twins in a healthcare or biomedical context,
2. explicitly considered *multi-scale integration* (i.e., involvement of at least two biological or clinical layers among molecular, cellular, tissue/organ, clinical, behavioral, or environmental levels), or
3. made substantial contributions to at least one of the following domains:
  – conceptual foundations of digital twins in medicine,
  – technical architectures enabling multi-scale modeling,
  – clinically evaluated applications of digital twins,
  – ethical, regulatory, or data-governance challenges specific to healthcare digital twins.

Original research articles, systematic or narrative reviews, methodological papers, perspectives, and policy papers published in peer-reviewed journals and written in English were considered.

#### Exclusion criteria

Articles were excluded if they:

− focused exclusively on industrial, manufacturing, or engineering digital twins without healthcare translation,
− described single-scale models only (e.g., imaging-only or EHR-only systems without multi-scale integration),
− were conference abstracts, editorials without methodological contribution, or non–peer-reviewed sources.

#### Screening procedure

A total of 184 records were initially identified after duplicate removal. Titles and abstracts were screened to assess relevance to multi-scale digital twins in healthcare. Articles passing this stage underwent full-text review.

#### Rationale for final selection

Twenty articles were retained for the core synthesis (Table 1) because they jointly:

− covered all major dimensions of multi-scale digital twins (conceptual, technical, clinical, ethical, and regulatory),
− explicitly addressed integration across biological and clinical layers,
− represented diverse methodological paradigms (mechanistic modeling, machine learning, causal inference, federated learning, hybrid architectures), and
− provided sufficient methodological depth to serve as reference frameworks.

**Table 1:** Summary of references included.

References	Type of article	Main focus	Main results/findings	Key contribution to MSDT	Field/domain	Section in review	Data layers addressed
(1)	Perspective	Complexity and emergent properties in digital twins	Advocates for complex systems modeling in medicine	Defines theoretical basis for MSDTs	Systems medicine	Conceptual foundations	Molecular, cellular, tissue/organ, clinical
(2)	Review	Personalized vs. population health digital twins	Dual application model	Broadens MSDTs to public health impact	Public health	Introduction, conceptual foundations	Clinical, behavioral, environmental
(15)	Perspective	Biospecimen digital twins	Promotes omics-driven modeling	Integrates multi-omics in MSDTs	Omics/biobanking	Conceptual foundations	Genomics, proteomics, metabolomics, spatial omics
(21)	Methodology	Causal inference in ML for healthcare	Introduces counterfactual frameworks	Causal reasoning for MSDT predictions	AI/causal inference	Technical architecture	Clinical
(24)	Review	Multimodal machine learning	Fusion strategy taxonomy	Basis for data integration in MSDTs	AI/ML	Technical architecture	Molecular, imaging, clinical
(25)	Review	Deep multimodal learning	Early vs. late fusion comparison	Refinement of multimodal learning for MSDTs	AI/ML	Technical architecture	Molecular, imaging, clinical
(20)	Review	Graph Neural Networks in bioinformatics	Survey of GNNs in health data	Suggests GNNs for multi-scale integration	AI/graph modeling	Technical architecture	Molecular, cellular, tissue
(9)	Methodology	Generative adversarial networks (GANs)	Simulating counterfactual outcomes	Synthetic patient trajectories for MSDTs	AI/generative models	Technical architecture	Clinical, behavioral
(28)	Original Research	Stochastic differential equations in pharma	SDEs to model biological variability	Introduces randomness and noise into MSDTs	Mathematical Modeling	Technical architecture	Cellular, organ
(8)	Original Research	Cardiac digital twins	Heart model simulation	First clinical cardiac MSDT examples	Cardiology	Clinical applications	Cellular, tissue, organ, clinical
(29)	Research	Mutational processes in cancer	Mapping mutational signatures	Genomic modeling basis for oncology MSDTs	Cancer genomics	Clinical applications	Genomics, molecular
(30)	Perspective	Genomic privacy risks	Highlight re-identifiability risks	Emphasizes ethics in MSDTs	Data ethics	Ethical challenges	Molecular
(13)	Perspective	Federated learning for healthcare	Proposes privacy-preserving ML	Critical for MSDTs data governance	AI/privacy	Ethical challenges	Clinical, behavioral
(31)	Research	Bias propagation in healthcare AI	Identifies systemic bias	Equity and fairness control in MSDTs	AI/ethics	Ethical challenges	Clinical, demographic
(32)	Review	Explainable AI (XAI) methods	Survey on XAI approaches	Improves MSDT transparency	AI explainability	Ethical challenges	Clinical
(50)	Research	AI breast cancer screening	International evaluation of AI	Reliability for clinical MSDTs validation	Digital health	Strategic roadmap	Imaging, clinical
(51)	Policy	GA4GH data standards	Policies for genomics interoperability	Foundation for MSDT cross-system integration	Genomics standards	Strategic roadmap	Genomics, transcriptomics, proteomics
(12)	Review	Physics-informed machine learning	New hybrid modeling paradigm	Mechanistic plus AI modeling for MSDTs	AI/mathematical modeling	Strategic roadmap	Molecular, organ
(52)	Perspective	In silico clinical trials	Virtual patient simulation	Twin-based clinical trial design	Clinical Research Innovation	Strategic roadmap	Organ, clinical
(14)	Perspective	Ethics of AI in healthcare	Ethical design principles for AI	Embedding ethics-by-design in MSDTs	Digital ethics	Strategic roadmap	Molecular, clinical, behavioral, environmental

These 20 articles constitute the structural backbone of the review. Additional references were incorporated throughout the manuscript to support specific technical methods, clinical domains, and regulatory aspects.

Thus, the review follows a purposeful and theory-driven selection strategy rather than an exhaustive systematic review approach, aiming to synthesize foundational and representative contributions rather than maximize numerical coverage.

**Figure 1:**
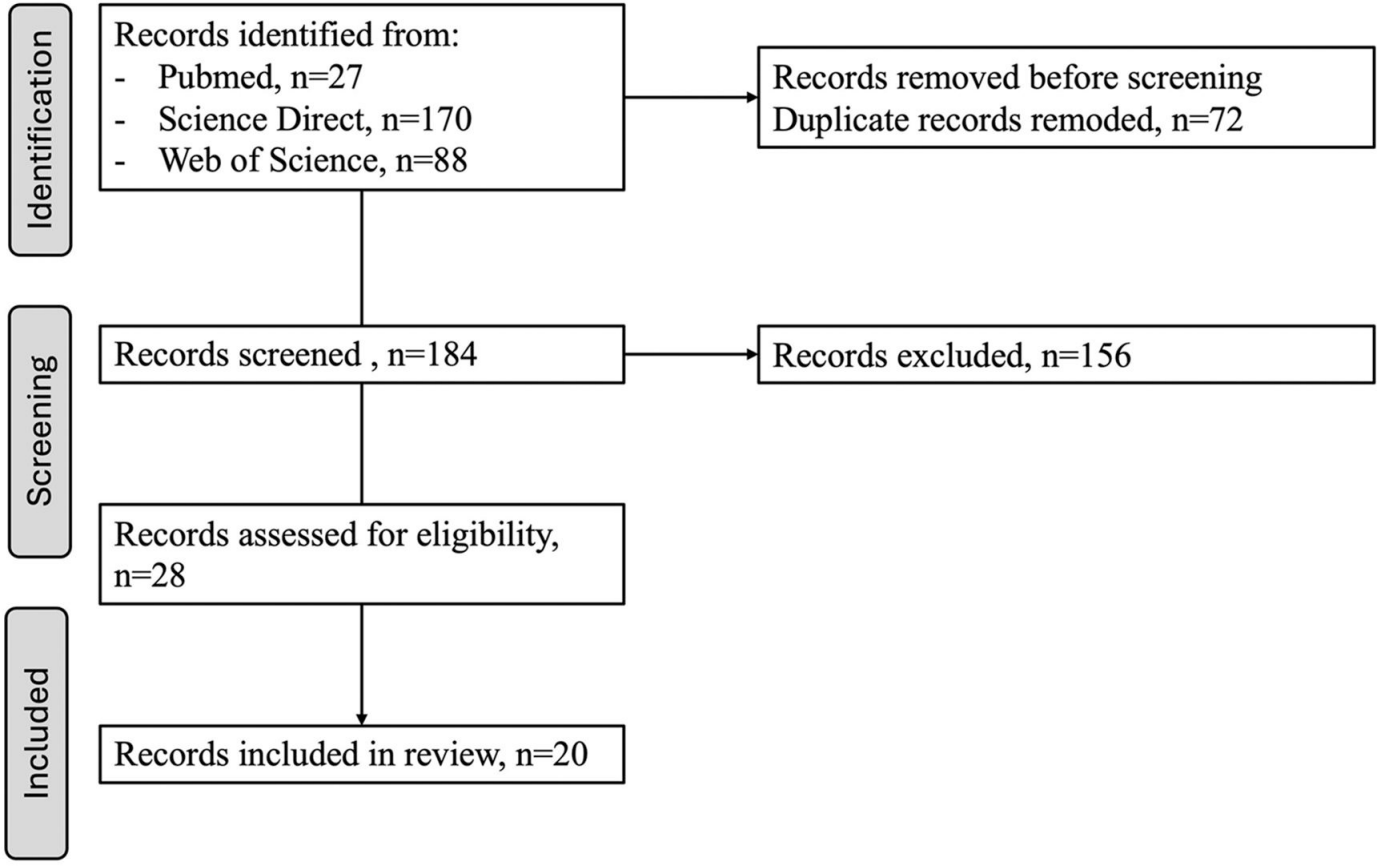
Flowchart.

The screening process is summarized in Figure 1.

### Conceptual foundations of multi-scale digital twins

MSDTs embody the principle that health and disease processes unfold across interconnected biological layers, each governed by distinct but interdependent dynamics. Building robust MSDTs requires a deep understanding of these layers and their interactions.

#### Defining “multi-scale” in biological systems

Biological systems are inherently hierarchical. Molecular events, such as gene mutations or protein misfolding, influence cellular functions, which in turn affect tissue organization, organ physiology, and ultimately whole-organism health. Simultaneously, behavior, environment, and societal factors feed back into molecular and physiological states (1, 2). Therefore, “multi-scale” refers not only to different spatial scales (e.g., nanometers to meters) but also to different informational and temporal scales (e.g., gene expression fluctuations over seconds vs. lifestyle changes over decades).

#### Hierarchical data layers: from genes to environment

**Figure 2:**
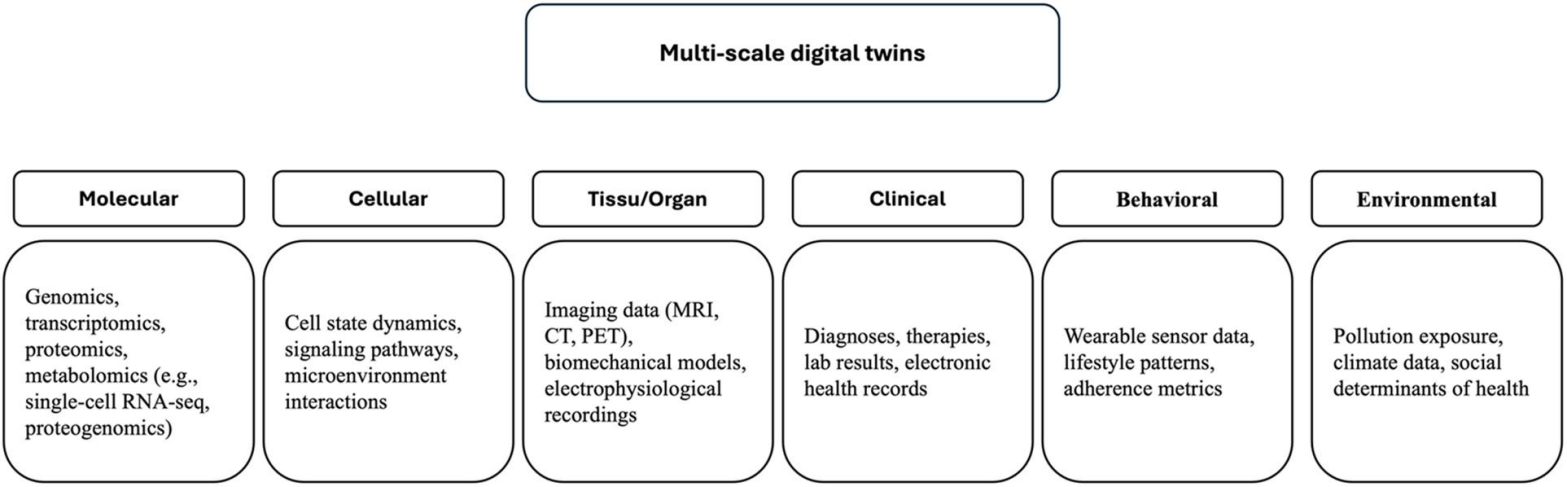
Hierarchical structure of data layers integrated into multi-scale digital twins.

An effective MSDT must incorporate and synchronize data across multiple layers (Figure 2) (15, 16). Each layer provides partial, complementary information. For instance, a genetic predisposition to diabetes may manifest differently depending on a patient's diet, physical activity, and socioeconomic context (7, 17). Integrating these scales enhances predictive accuracy and enables scenario simulation beyond what single-layer models can achieve.

#### Interdependencies and emergent properties across scales

**Figure 3:**
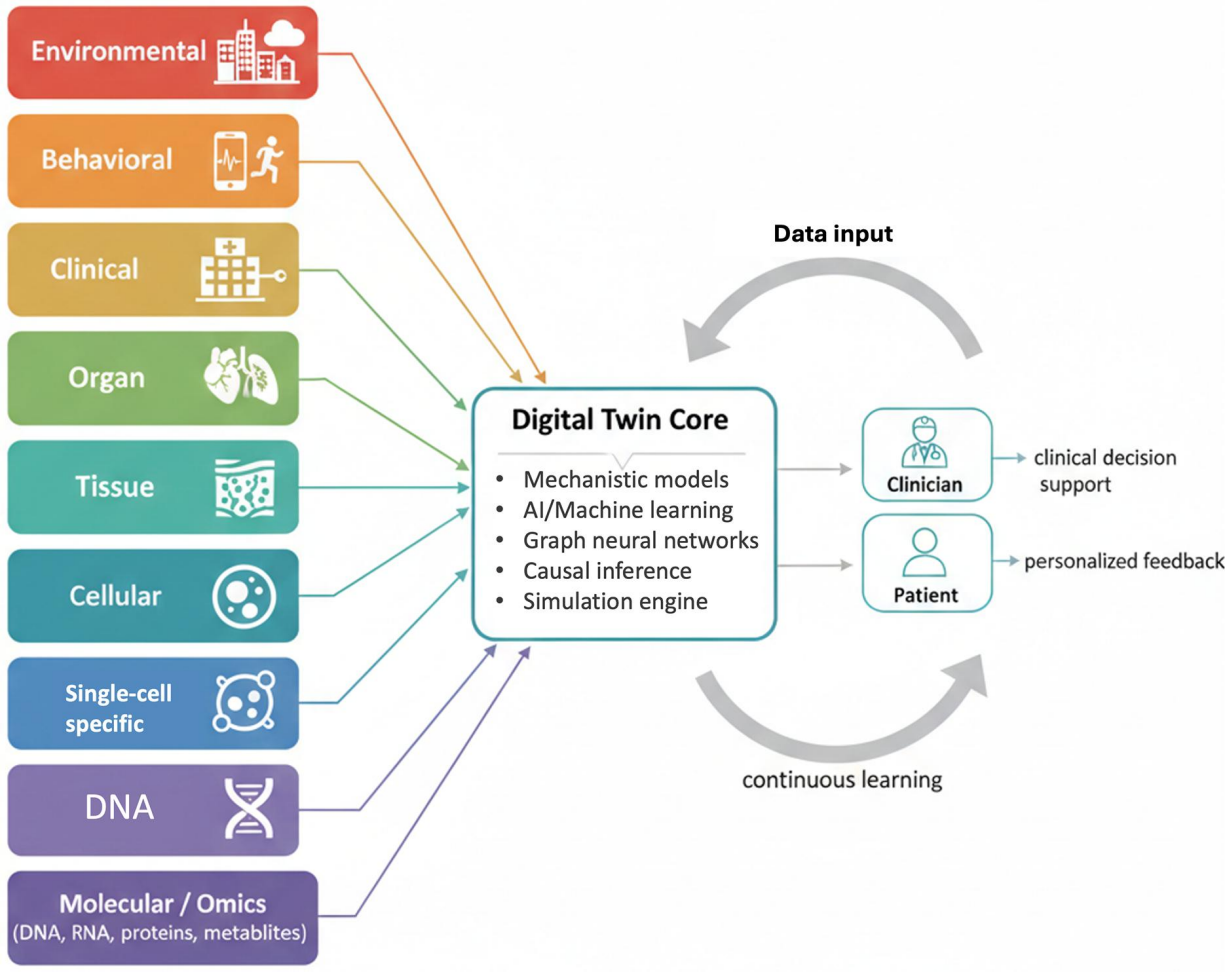
Conceptual architecture of a multi-scale digital twin for personalized medicine.

Crucially, biological phenomena exhibit emergent properties, namely system-level behaviors that cannot be predicted by analyzing individual components in isolation. Such properties arise from nonlinear interactions, feedback loops, and network effects across molecular, cellular, tissue, and organismal scales (1, 18) (Figure 3).

For example, heart failure cannot be fully explained by cardiomyocyte dysfunction alone but emerges from complex couplings between myocardial structure, electrophysiology, neurohormonal regulation, and hemodynamics. Similarly, cancer progression results from the interplay between genetic alterations, tumor microenvironment, immune dynamics, and systemic factors (8, 19).

From a theoretical standpoint, emergence is a defining feature of complex systems and networked multi-scale models, where macroscopic behaviors arise from collective interactions among heterogeneous microscopic entities rather than from simple additive effects (18). This property is central to the motivation for multi-scale digital twins: without explicit modeling of cross-scale interactions, digital twins risk remaining reductionist and unable to reproduce clinically relevant dynamics.

Consequently, MSDTs must represent not only individual biological layers but also their interdependencies. Techniques such as multi-modal graph representations, hybrid mechanistic–statistical models, and causal inference frameworks are essential to capture these emergent phenomena and to enable robust simulation of disease trajectories and intervention scenarios (20, 21).

### Technical architecture for multi-scale integration

Building a functional multi-scale digital twin (MSDT) first requires the definition of a coherent *multi-level and multi-layer system model* of the biological entity under study. Before considering data availability or fusion strategies, it is necessary to specify the relevant biological scales (molecular, cellular, tissue, organ, clinical, behavioral, environmental), their state variables, and the causal and functional interactions linking them (1, 18).

From a systems and network medicine perspective, this modeling step is foundational: without an explicit representation of cross-scale dependencies and feedback loops, subsequent data integration risks producing high-dimensional but mechanistically inconsistent models (22). Recent work in network medicine emphasizes that disease phenotypes emerge from perturbations of interconnected molecular and physiological networks, rather than from isolated components, reinforcing the need for structured, multi-layer modeling frameworks prior to data-driven learning (22).

More broadly, the theory of complex biological systems provides formal tools for constructing such hierarchical models, including multi-layer networks, dynamical systems, and stochastic processes that capture both within-scale dynamics and cross-scale coupling (18, 23). These approaches offer principled ways to represent emergence, nonlinearity, and system-level behavior, which are central to the objectives of MSDTs.

Only once this modeling scaffold is established does multi-modal data fusion become the next critical challenge. At that stage, heterogeneous data streams, such as genomics, proteomics, imaging, clinical variables, behavioral metrics, and environmental exposures, can be mapped onto the predefined model structure and integrated using early-, late-, or hybrid-fusion strategies (24, 25).

To manage these fused datasets, advanced artificial intelligence (AI) methods are essential. Graph Neural Networks (GNNs) have emerged as a particularly powerful tool for representing complex biological and clinical networks, where nodes can represent genes, tissues, comorbidities, or lifestyle factors, and edges capture functional relationships (26). GNNs allow the model to reason over relationships rather than isolated features, an essential capacity for multi-scale biological systems.

Beyond graph models, causal inference techniques are crucial. Traditional predictive models often capture correlations rather than causation, which limits their clinical utility for decision support. Structural causal models, counterfactual modeling, and potential outcome frameworks provide the mathematical foundation for predicting “what would happen if” scenarios, a central feature of effective MSDTs (21).

Furthermore, reinforcement learning (RL) plays a key role when MSDTs are used for sequential decision-making, such as optimizing chronic disease management over time. RL agents learn optimal treatment policies by interacting with the simulated twin environment, continuously updating strategies as patient data evolves (27). Particularly in dynamic and long-term conditions like diabetes or heart failure, RL-enabled twins could personalize therapeutic adjustments in real time.

Generative models such as Generative Adversarial Networks (GANs) and diffusion models further enrich MSDT capabilities. They can simulate synthetic yet realistic patient trajectories, predict rare adverse events, or augment sparse datasets by generating plausible unobserved scenarios (9). These generative approaches help bridge the gap between incomplete observational data and the full complexity of human health dynamics.

For dynamic simulation, multi-scale modeling frameworks are essential. Agent-based models represent entities like cells, immune responses, or patient behaviors as autonomous agents, capable of interacting with one another according to defined rules. These models are particularly valuable for studying emergent phenomena such as tumor microenvironment evolution or epidemic spread. Meanwhile, mechanistic multi-scale models integrate molecular dynamics (e.g., signaling pathways), organ function (e.g., cardiac electrophysiology), and systemic responses (e.g., hemodynamics), often using systems of differential equations.

Because biological systems are stochastic by nature, stochastic differential equations (SDEs) offer a mathematically rigorous way to capture random fluctuations in health status over time (28). For example, blood glucose dynamics in diabetes are influenced by intrinsic metabolic noise, environmental variability (e.g., meals, stress), and behavioral randomness (e.g., exercise), all of which can be elegantly captured using SDEs.

Ultimately, the technical architecture of MSDTs must balance three core imperatives: richness (capturing cross-scale interactions in high resolution), robustness (handling noise, missingness, and uncertainty), and interpretability (allowing clinicians to understand and trust the model outputs). Architectures that combine mechanistic knowledge (from biology and physics) with flexible AI components will likely outperform purely data-driven or purely theoretical models.

Building such systems requires not only technical innovation but also an interdisciplinary commitment to open data standards, scalable computational infrastructure, and clinical validation. Only through this integration of techniques and philosophies can multi-scale digital twins transition from conceptual promise to clinical reality, driving the future of personalized medicine.

### Clinical applications and use cases

The implementation of MSDTs unlocks transformative possibilities across a wide spectrum of clinical fields (Table 2). By integrating diverse biological, clinical, behavioral, and environmental data, MSDTs enable unprecedented personalization of diagnostics, prognostics, and therapeutics.

**Table 2:** Examples of clinically relevant digital twins reported in literature.

Clinical domain	System/study	Multi-scale components integrated	Clinical use case	Level of evidence/implementation	References
Cardiology: Cardiac Electrophysiology Twin	Personalized cardiac digital twins (ventricular EP from ECG + MRI)	Anatomical models, electrophysiology simulation, ECG inversion	Non-invasive arrhythmia localization and ablation planning	Proof-of-concept workflow performed on clinical images with feasible computation within clinical timeframes (<4 h)	(19)
Oncology: High-Grade Glioma Predictive Twin	Data-driven predictive tumor twin (multi-objective Bayesian calibration)	Mechanistic tumor growth + MRI data + treatment response simulation	Personalized radiotherapy optimization (risk-aware regimens)	Retrospective in-silico cohort evaluation demonstrating personalized optimal regimens vs. SOC	(53)
Diabetes: Type 1 Diabetes Twins (Systematic Review)	Collection of DT models focused on glucose-insulin dynamics	Mathematical glucose–insulin models + patient metadata	Simulation of glucose control and therapy response	Systematic characterization of 8 T1D DT methodologies; limited multi-scale replication beyond metabolic dynamics	(54)
Diabetes: Digital Twin Intervention Outcomes (Type 2)	Digital twin-driven precision treatment program	CGM, activity, nutrition, AI interpretation	One-year glycemic control and medication reduction in T2D	Retrospective observational study showing significant improvements in HbA1c and metabolic markers	(55)
Neurology: Virtual Brain Twin (Epilepsy)	Personalized virtual brain model twin	Structural MRI + tractography + EEG/SEEG dynamics	Estimation of epileptogenic zones for surgical planning	Retrospective cohort modeling demonstrating concordance with clinical data and surgical outcomes	(56)
Healthcare Systems/Precision Health	Review of digital twin frameworks in clinical settings	Multiple modalities: imaging, sensor data, physiological inputs	Broad care personalization (prediction, management, workflow)	Systematic overview of claimed digital twins with classification of simulation vs. monitoring vs. research models	(57)
Urological Oncology (Editorial/Perspective)	Urological oncology digital twins	Conceptual modeling of patient + tumor dynamics	Treatment planning support in urology oncology	Perspective summarizing potential DT applications; not yet quantitative clinical data	(58)

In personalized oncology, MSDTs can integrate genomic profiles (e.g., mutational burden, microsatellite instability) (29), transcriptomic signatures (e.g., immune infiltration markers), imaging phenotypes (e.g., radiomics), and treatment histories to simulate tumor evolution under various therapeutic strategies. By modeling both tumor-intrinsic factors and the tumor microenvironment, MSDTs can predict individual response trajectories to chemotherapy, immunotherapy, or targeted agents. For instance, a patient's likelihood of developing immune-related adverse events during checkpoint inhibitor therapy could be anticipated through twin simulations, allowing for proactive management strategies.

In cardiometabolic diseases, the multi-scale integration of genetic predispositions (e.g., lipid metabolism variants), lifestyle behaviors (e.g., diet, exercise), physiological parameters (e.g., blood pressure, glycemic control), and imaging data (e.g., coronary CT angiography) enables MSDTs to forecast disease progression and optimize intervention timing (8). For example, a digital twin of a patient at risk for atherosclerosis could simulate the impact of different lifestyle modifications, pharmacological treatments (e.g., statins, PCSK9 inhibitors), or procedural interventions (e.g., stenting) on future cardiovascular events. Furthermore, continuous real-time updates through wearable data can recalibrate risk predictions dynamically, enhancing secondary prevention strategies.

Nevertheless several challenges remain. Clinical validation of MSDTs must be rigorously pursued through prospective trials. Interoperability between data systems, especially integrating real-world data from heterogeneous sources, must be standardized. Ethical concerns, including data ownership, informed consent for model use, and ensuring equitable access to these technologies, require proactive governance frameworks (14). By simulating complex biological systems across scales, MSDTs promise not only to predict individual health trajectories but also to reshape them through optimized, dynamic, and personalized interventions. Their implementation marks a critical step towards realizing the vision of truly individualized, preventive, and precision healthcare.

#### Why clinically deployable MSDTs remain rare: the model-first bottleneck

Despite the increasing number of “digital twin” applications, fully multi-scale digital twins remain uncommon in real clinical environments. A key reason is that the first and most fundamental challenge is to build a working multi-level model of the biological system, with explicitly defined state variables, cross-scale coupling mechanisms, and clinically actionable outputs (1, 22, 23). In practice, multi-modal data availability and fusion are secondary challenges that can only be addressed once the model structure and its identifiability constraints are established.

From a technical standpoint, the major obstacles to multi-scale digital twin design include:

(i) cross-scale coupling design, i.e., specifying how molecular and cellular dynamics causally influence organ-level phenotypes and clinical trajectories;
(ii) parameter identifiability and observability, since multi-scale models often contain latent states and parameters that cannot be uniquely inferred from available clinical measurements;
(iii) uncertainty propagation, where measurement noise, missingness, and model mismatch at one layer can amplify errors at higher layers;
(iv) computational tractability, as coupled multi-scale simulations can be too expensive for iterative calibration or real-time updating; and
(v) validation strategy, as multi-scale twins must be evaluated not only for predictive accuracy but also for counterfactual fidelity and treatment-response simulation (1, 22, 23).

Importantly, many clinically mature digital twins currently deployed in practice focus on one dominant scale (e.g., organ-level biomechanics or physiology) but may not explicitly claim multi-scale features. These systems can be viewed as proto-MSDTs that provide validated, clinically grounded cores onto which additional biological, behavioral, and environmental layers can progressively be integrated.

### Ethical, regulatory, and infrastructural challenges

The development and deployment of MSDTs in healthcare introduce profound ethical, regulatory, and infrastructural challenges that must be proactively addressed to ensure responsible innovation and equitable access.

A primary ethical concern is data privacy and ownership. MSDTs rely on extensive, longitudinal, and multi-source data, including sensitive genomic, behavioral, and environmental information. The aggregation and analysis of such data raise significant risks of re-identification, even when anonymized datasets are used (30). Traditional privacy protection measures, such as de-identification, may prove insufficient. Therefore, new governance models are required, emphasizing dynamic consent, secure federated learning architectures, and patient-controlled data access mechanisms (13).

Closely related is the issue of algorithmic fairness and bias propagation. Health disparities embedded in historical datasets, due to systemic inequities, can be amplified if not explicitly mitigated during the development of MSDTs. For example, underrepresented populations may experience less accurate predictions or suboptimal simulated interventions (31). Fairness audits, subgroup performance evaluations, and bias correction algorithms must become standard practice to ensure equitable clinical utility across demographic groups.

Transparency and explainability represent further critical dimensions. Clinicians and patients must be able to understand the rationale behind MSDT-generated recommendations, especially in high-stakes contexts like oncology or critical care. Techniques such as counterfactual explanations, SHAP (SHapley Additive exPlanations) values, and interpretable surrogate models should be systematically embedded within MSDT architectures (32). Transparency is not merely a technical feature; it is foundational to trust, informed consent, and effective shared decision-making.

On the regulatory front, current frameworks are ill-adapted to the complexities of dynamic, self-adaptive digital twins. Unlike static medical devices or predefined clinical decision support systems, MSDTs continuously evolve based on new data streams and reinforcement learning feedback loops. Regulatory bodies such as the FDA and EMA are beginning to explore adaptive approval pathways, but clear guidance on validation, risk categorization, and post-deployment monitoring for MSDTs remains urgently needed.

Validation itself poses major challenges. Conventional randomized controlled trials may be insufficient to assess continuously updating MSDTs. Alternative approaches, such as digital twin randomized simulations, real-world evidence collection, and adaptive trial designs, must be developed and accepted by regulatory authorities to ensure rigorous yet flexible evaluation (33).

Infrastructurally, interoperability is a bottleneck. MSDTs require seamless data integration across electronic health records, wearable sensors, genomic databases, and environmental monitoring systems. Yet, data silos, incompatible standards, and proprietary formats persist. The adoption of universal interoperability frameworks such as HL7 FHIR (Fast Healthcare Interoperability Resources) and OMOP CDM (Observational Medical Outcomes Partnership Common Data Model) is essential to allow scalable and reproducible MSDT development (34).

Moreover, the computational demands of MSDTs, particularly for real-time simulation, reinforcement learning, and high-dimensional data fusion, necessitate robust, secure, and scalable infrastructures. Cloud-based architectures with strong encryption, federated learning platforms, and edge computing solutions will be critical to meet latency and privacy requirements (35).

Then, social and cultural factors must not be overlooked. Patient acceptance of MSDTs will depend not only on technical performance but also on perceived benefits, risks, and alignment with individual values. Co-design approaches involving patients, clinicians, ethicists, and technologists are crucial to ensure that MSDTs serve humanistic goals rather than merely technological advancement (36).

While multi-scale digital twins hold transformative potential, their responsible deployment demands a comprehensive ethical, regulatory, and infrastructural ecosystem. Proactively addressing these challenges will be pivotal in ensuring that MSDTs fulfill their promise of enhancing health outcomes, reducing disparities, and fostering trust in an increasingly data-driven era of medicine.

### International collaboration, data sovereignty, and regulatory harmonization

While interdisciplinary collaboration is fundamental to the development of multi-scale digital twins, global data diversity is equally critical to ensure model robustness, fairness, and clinical generalizability across populations, healthcare systems, and socio-environmental contexts (37). MSDTs trained exclusively on geographically or ethnically homogeneous datasets risk limited external validity and the amplification of structural biases (38).

However, international collaboration in this domain is constrained by heterogeneous data sovereignty and privacy regulations, including the General Data Protection Regulation (GDPR) in the European Union, the Health Insurance Portability and Accountability Act (HIPAA) in the United States, the Personal Data Protection Act (PDPA) in Singapore, and the Chinese Cybersecurity Law and Data Security Law (39–41). These frameworks impose differing requirements regarding data localization, cross-border transfer, consent, and secondary use, complicating the construction of globally integrated digital twin infrastructures.

Several technical and governance mechanisms can enable meaningful international collaboration without violating sovereignty constraints. Federated learning architectures allow MSDT models to be trained across distributed datasets while keeping sensitive patient-level data within national or institutional boundaries (13, 42). Only encrypted model parameters or gradients are exchanged, substantially reducing legal and ethical risks associated with cross-border data transfer. Complementary privacy-preserving techniques, including secure multi-party computation, homomorphic encryption, and differential privacy, further mitigate re-identification risks in international model development (13, 43, 44).

At the organizational level, emerging concepts such as data trusts, data cooperatives, and international research data spaces provide legal and contractual frameworks to govern access rights, usage limitations, liability, and benefit sharing across jurisdictions (45). In parallel, regulatory sandboxes operated by health authorities offer controlled environments for testing cross-border digital health and AI systems under real-world conditions while maintaining regulatory oversight (46).

From a strategic perspective, harmonization efforts should focus not on uniform legislation, which is politically unrealistic, but on interoperability of compliance. This includes standardized consent models, metadata schemas describing regulatory constraints, machine-readable data-use policies, and international certification mechanisms for privacy-preserving digital twin platforms (47–49).

Ultimately, scalable and ethically responsible MSDTs will require a dual foundation: local compliance with national data sovereignty laws and global coordination through technical standards and governance frameworks. Without such international alignment, digital twins risk reinforcing existing data monopolies and regional biases rather than enabling truly universal personalized medicine.

### Strategic roadmap and recommendations

To fully realize the transformative potential of MSDTs in healthcare, a coordinated and strategic roadmap is essential. This roadmap must span data infrastructure, methodological innovation, regulatory alignment, and interdisciplinary collaboration.

There is a critical need to build multi-scale biobanks and longitudinal cohorts. Traditional clinical cohorts often lack the temporal depth, biological breadth, and environmental diversity required for MSDTs. New initiatives must integrate genomic, proteomic, imaging, behavioral, and environmental data collected over time, ideally beginning early in life and continuing across the lifespan (50). Initiatives like the UK Biobank and the All of Us Research Program provide templates but must be extended to encompass dynamic, multi-modal, and ethnically diverse populations.

Promoting interoperability and data standards is crucial. Standardization initiatives such as HL7 FHIR for clinical data and GA4GH (Global Alliance for Genomics and Health) frameworks for omics data should be mandated or incentivized to ensure seamless integration into MSDT platforms (51). Interoperability is not merely technical; it is foundational for collaboration, reproducibility, and scalability.

Methodological innovation must prioritize hybrid modeling approaches. Combining mechanistic models grounded in biology with machine learning models enables both interpretability and predictive flexibility (12). Emphasis should be placed on developing hybrid AI systems that can simulate causal relationships, handle uncertainty, and adapt over time as new data become available.

Cross-disciplinary consortia should be established to bridge gaps between clinicians, computer scientists, systems biologists, ethicists, and policymakers. Initiatives such as the European Virtual Human Twin project exemplify the power of collaborative efforts. Institutional support and funding mechanisms must favor interdisciplinary, multi-institutional partnerships over soiled research (52).

Regulatory frameworks must adapt to the dynamic nature of MSDTs. Regulators should develop new categories for continuously learning systems, establishing guidelines for safe updates, ongoing validation, and transparent reporting of model evolution. Regulatory sandboxes, controlled environments where innovative technologies can be tested under regulatory supervision, should be expanded for MSDTs.

Sixth, ethics and governance must be embedded from the outset. MSDT development should follow principles of responsible innovation, including anticipatory ethics, participatory design involving patients, and proactive bias mitigation strategies (14). Governance frameworks should ensure not only data protection but also the equitable distribution of benefits derived from digital twin technologies.

Investment in education and capacity building is vital. Clinicians must be trained to interpret, validate, and integrate MSDT outputs into clinical decision-making. Similarly, engineers and data scientists must gain deeper understanding of medical contexts and ethical responsibilities. Dedicated curricula at the intersection of AI, systems medicine, and bioethics should be developed to cultivate the next generation of MSDT researchers and practitioners.

### Limitations

Very few MSDT implementations have undergone large-scale clinical validation or prospective evaluation in real-world settings. Consequently, some proposed applications discussed herein are extrapolations based on technological potential rather than proven clinical efficacy. The integration of multi-scale data (from omics to clinical and behavioral layers) poses significant technical, infrastructural, and interoperability challenges. Many reviewed studies focus on single-scale or bi-scale models rather than fully integrated frameworks. Therefore, the generalizability of current methodologies to true multi-scale clinical practice remains uncertain. Because the structured narrative synthesis prioritized papers explicitly discussing multi-scale digital twins, some clinically deployed single-domain digital twins that do not self-identify as “multiscale” may be underrepresented in the clinical examples. This work therefore interprets current clinically mature twins as “proto-MSDTs” and discusses the technical requirements needed to extend them toward fully integrated multi-scale architectures.

## Conclusion

MSDTs offer a transformative approach to personalized medicine by integrating data across molecular, clinical, behavioral, and environmental levels. By simulating complex biological systems dynamically, MSDTs enable more precise prediction, prevention, and treatment strategies tailored to individual patients. However, significant challenges remain regarding data integration, clinical validation, ethical governance, and regulatory adaptation. Addressing these barriers through interdisciplinary collaboration and responsible innovation is essential to fully realize the potential of MSDTs in future healthcare.
